# The Role of Prognostic Nutritional Index in UTI Susceptibility Among Female Type 2 Diabetic Patients

**DOI:** 10.1155/jdr/6890754

**Published:** 2025-12-09

**Authors:** Chao-Yin Lu, Qiao Liu, Ping Feng, Han-Ying Liu, Yan-Mei Shan, Meng-Die Chen

**Affiliations:** ^1^ Department of Endocrinology, Taizhou Central Hospital (Taizhou University Hospital), Taizhou, Zhejiang Province, China, tzc.edu.cn; ^2^ Department of Nephrology, Taizhou Central Hospital (Taizhou University Hospital), Taizhou, Zhejiang Province, China, tzc.edu.cn

**Keywords:** diabetes mellitus, fasting blood glucose, prognostic nutritional index, risk factor, urinary tract infection

## Abstract

**Background:**

Compared with individuals without diabetes, patients with diabetes have a higher susceptibility to urinary tract infection (UTI), and the infection is often more severe. Women are particularly vulnerable compared with men. This study attempted to explore predicted indicators and risk factors of the occurrence of UTI in women with Type 2 diabetes mellitus (T2DM).

**Aim:**

The aim of this study is to identify bacterial distribution and antimicrobial resistance patterns and to assess clinical risk factors for UTI in women with T2DM.

**Methods:**

A retrospective study was carried out on 136 patients with diabetes mellitus hospitalized in the Endocrinology Department of Taizhou Central Hospital (Taizhou University Hospital). The levels of fasting blood glucose (FBG), glycosylated hemoglobin A1c (HbA1c), hemoglobin, peripheral blood leukocyte count, neutrophil count, monocyte count, C‐reactive protein, and urine pH were collected. Demographic data, physical examination data, and medical history were also collected. Urine culture was performed using the Autof ms1000, while antimicrobial susceptibility testing was conducted with the VITEK 2.

**Results:**

One hundred thirty‐six patients aged 60.49 ± 13.02 years old were enrolled in our study, and a total of 30 patients had positive urine cultures. Height, marital status, HbA1c, FBG, prognostic nutritional index (PNI), urine pH, and peripheral blood leucocyte‐to‐monocyte ratio were associated with UTI (*p* < 0.05). Multivariable logistic regression analysis showed that marital status (odds ratio = 6.33, 95*%*CI = [1.06 ~ 37.82], *p* = 0.043), FBG (odds ratio = 1.26, 95*%*CI = [1.08 ~ 1.46], *p* = 0.004), and PNI (odds ratio = 0.89, 95*%*CI = [0.81 ~ 0.99], *p* = 0.025) were the influencing factors for UTI. *Escherichia coli* (14/30, 46.7%) was the most common pathogen, and the susceptibility rate of levofloxacin to *E. coli* was low (1/14, 7.1%).

**Conclusion:**

The levels of PNI, FBG, and marital status were identified as risk factors for UTI in women with T2DM. *E. coli* was the most common causative agent.

## 1. Introduction

Among adults aged 20–79 years old, an estimated 536.6 million people (uncertainty interval, 424.2–612.3 million) were diagnosed with diabetes in 215 countries and territories, corresponding to a global prevalence of 10.5% (uncertainty interval, 8.3%–12.0%). China was one of the countries with the largest number of diabetics [1]. A nationwide cross‐sectional investigation covering 31 provinces in mainland China and including 75,880 participants reported a weighted prevalence of diabetes based on ADA diagnostic criteria of 12.8% (95% CI: 12.0%–13.6%) [2]. A study enrolled 3652 Chinese patients with Type 2 diabetes mellitus (T2DM) and found that 409 (11.2%) patients had a urinary tract infection (UTI) [3]. Evidence further indicates that compared with individuals without diabetes, patients with diabetes have a higher susceptibility to UTI [4–6]. Moreover, UTI was more severe in diabetic patients, as pathogens are more likely to develop drug resistance, and prognosis was worse [7].

Multiple mechanisms caused the increased risk of UTI in diabetics [4]. Almost half of people with diabetes have bladder dysfunction. Bladder dysfunction was related to decreased sensation of bladder filling and poor contractility, finally leading to increased residual urine and UTI [8]. Glucosidase was associated with the occurrence of UTI; however, no clear boundary was found [9]. Type 1 fimbriae played a crucial role in the occurrence of UTI, and their adhesion to uroepithelial cells was positively correlated with glycosylated hemoglobin A1c (HbA1c) [10]. Moreover, hyperglycemia was associated with a weakened immune system, characterized by decreased mobilization, chemotaxis, and phagocytic activity of multinucleated leukocytes [11].

The excessive and inappropriate use of antibiotics often results in antibiotic resistance. The development of bacterial resistance tends to cause more severe infections, resulting in longer treatment times and a greater burden of treatment costs [12]. Therefore, our study was undertaken to assess the clinical bacterial spectrum and antimicrobial susceptibility patterns in T2DM patients with UTI treated at Taizhou Central Hospital (Taizhou University Hospital). Considering that women were more likely to develop UTI than men [5, 13–15], women were the subjects of our study. Moreover, the study was aimed at identifying predictive indicators and risk factors associated with UTI occurrence in women with T2DM.

## 2. Materials and Methods

In this retrospective study, data were collected from 146 female patients aged ≥ 18 years old with T2DM who were hospitalized in the endocrinology department of Taizhou Central Hospital (Taizhou University Hospital) between December 29, 2020, and December 22, 2021. T2DM was diagnosed based on the Diagnosis and Classification of Diabetes: Standards of Care in Diabetes‐2024 [16]. The exclusion criteria included suffering from serious illness, accepting antimicrobial drugs in the last 2 weeks, or pregnancy.

UTI was defined as the presence of at least 10^5^ cfu/mL bacteria detected in fresh midstream urine, and the presence of more than five leukocytes per high‐power field also indicated UTI. Multidrug‐resistant (MDR) bacteria were defined as being insensitive to at least three antibiotics.

Demographic data including marital status, age, and physical examination data, such as body mass index (BMI), weight, height, and blood pressure, as well as medical history including inpatient days and duration of diabetes, were collected. The information on sex was obtained by self‐report. The levels of fasting blood glucose (FBG), C‐reactive protein, HbA1c, hemoglobin, peripheral blood leucocyte count, monocyte count, neutrophil count, and urine pH were also collected on the second day after admission. The formula used included the following: prognostic nutritional index (PNI) = serum albumin (g/L) + 5 × lymphocyte count (10^9^/L).

Midstream clean‐catch urine samples were collected from all patients. Once elevated urinary leukocytes were detected, further urine culture and antimicrobial sensitivity tests were performed. Urine culture was performed using the Autof ms1000, while antimicrobial susceptibility testing was conducted with the VITEK 2. All operations were carried out by technicians from the department of laboratory medicine. Bacteriuria was defined as a colony count of ≥ 10^5^ CFU/mL for midstream clean‐catch urine. The detected strains were obtained from the Shanghai Clinical Laboratory Center. The gram‐positive bacteria were tested for drug sensitivity to the following drugs: penicillin G, levofloxacin, tigecycline, moxifloxacin, erythromycin, vancomycin, and linezolid. The gram‐negative bacteria were tested for drug sensitivity to the following drugs: cefuroxime, cefoxitin, cefepime, ceftazidime, levofloxacin, amikacin, cefoperazone sodium/sulbactam sodium, piperacillin sodium/tazobactam sodium, imipenem, and ertapenem. Meanwhile, fungus was tested for sensitivity to the following drugs: itraconazole, fluconazole, voriconazole, amphotericin B, and 5‐fluorocytosine.

Statistical Product and Service Solutions (SPSS) 26 was employed to conduct data analysis. The Shapiro–Wilk test, the Kolmogorov–Smirnov test, and Levene′s test were used to evaluate whether the two groups of quantitative data conformed to a normal distribution and had homogeneity of variance. Differences in quantitative variables were compared using a two‐sample *t*‐test and a Wilcoxon rank‐sum test. Differences in qualitative variables were compared using the *χ* ^2^ test and Fisher′s exact test. According to the results of the above analysis, the selected confounding factors were determined to be included in the binary logistic regression model (Enter method). All *p* values were two‐sided, and a *p* value < 0.05 was considered to be statistically significant. A receiver operating characteristic (ROC) curve was plotted to determine the optimal cut‐off for predicting UTI.

## 3. Results

A total of 136 patients aged 60.49 ± 13.02 years old were enrolled, and 30 patients had a positive urine culture. Age, inpatient stay, duration of diabetes, weight, BMI, and blood pressure were not related to the occurrence of UTI (*p* > 0.05, Table 1). Education background was also not related to the occurrence of UTI (*p* > 0.05, Figure 1). Patients with UTI were slightly taller than people without UTI (159.13 ± 5.34 cm vs. 156.51 ± 5.10 cm, *p* = 0.02, Table 1). Married patients were more likely to get UTI than nonmarried patients (including unmarried, divorced, and widowed patients, *p* = 0.040, Figure 1).

**Table 1:** Baseline characteristics for patients with or without urinary tract infection.

	**Patients with UTI (** **n** = 30 **)**	**Patients without UTI** ^**a**^ ** (** **n** = 106 **)**	**p** ** values**
Age (years old)	57.00 ± 12.94	61.47 ± 12.94	0.086
Age group (years old)			
< 55	13	26	0.066
55 or above	17	80	
Inpatients′ stay (days)	9.50 ± 3.67	8.18 ± 3.41	0.105
Inpatients stay group (days)			
< 7	4	33	0.064
7 or above	26	73	
Duration of diabetes (years)	11.34 ± 6.60	11.49 ± 7.52	0.902
Duration of diabetes group (years)			
< 10	8	37	0.511
10 or above	22	68	
Weight (kg)	59.31 ± 9.74	58.40 ± 9.76	0.853
Height (cm)	159.13 ± 5.34	156.51 ± 5.10	0.020^†^
BMI (kg/m^2^)	23.33 ± 3.01	23.79 ± 3.53	0.521
BMI group			
< 24	18	57	0.678
24 or above	12	48	
SBP (mmHg)	133.67 ± 19.73	133.52 ± 20.57	0.973
DBP (mmHg)	78.83 ± 10.63	78.74 ± 11.68	0.970
MBP (mmHg)	97.11 ± 11.82	97.00 ± 13.62	0.969

*Note:* Continuous variables are presented as the mean ± SD.

Abbreviations: BMI, body mass index; DBP, diastolic blood pressure; MBP, mean blood pressure; SBP, systolic blood pressure; UTI, urinary tract infection.

^a^The duration of diabetes and BMI data were not available for one patient with urinary tract infection.

^†^ *p* < 0.05.

Figure 1Proportion of patients with and without UTI stratified by educational level and marital status. (a) Separate proportion of patients with UTI and patients without UTI at different educational levels. (b) Separate proportion of patients with UTI and patients without UTI in different marital statuses. UTI, urinary tract infection.

**Figure:**
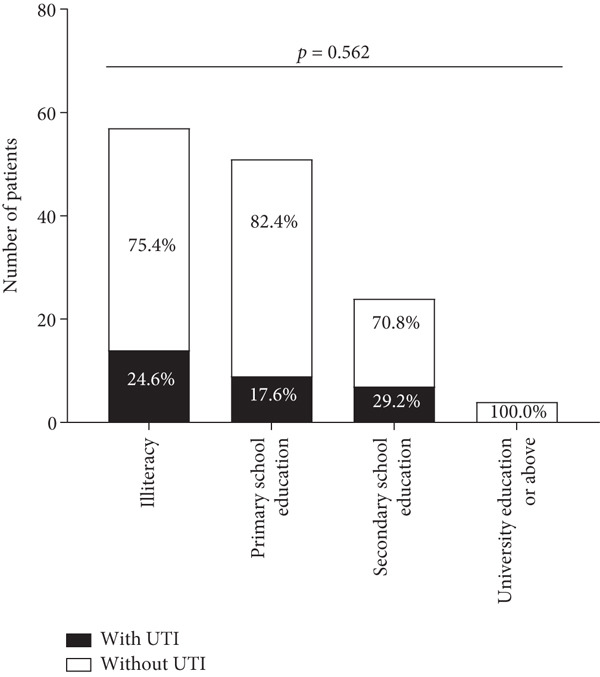
(a)

**Figure:**
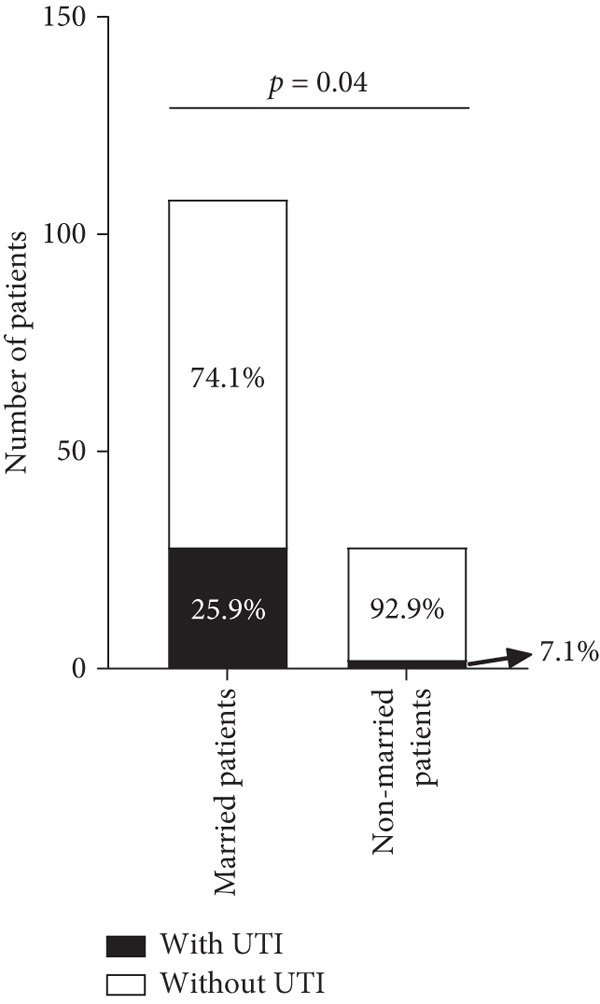
(b)

Patients with UTI had higher HbA1c (10.97*%* ± 2.57*%* vs. 9.61*%* ± 2.38*%*, *p* = 0.008) and FBG (12.02 ± 3.97 mmol/L vs. 8.19 ± 3.57 mmol/L, *p* < 0.001) than those without UTI but lower PNI (47.27 ± 7.08 vs. 50.13 ± 5.35, *p* = 0.018), urine pH (5.57 ± 0.59 vs. 5.75 ± 0.96, *p* = 0.018), and peripheral blood leucocyte‐to‐monocyte ratio (16.65 ± 3.94 vs. 19.09 ± 5.80, *p* = 0.040, Table 2). Laboratory results of peripheral blood leucocytes, neutrophils, and C‐reactive protein were not related to the occurrence of UTI (*p* > 0.05, Table 2). Multivariable logistic regression analysis showed that married status (odds ratio = 6.33, 95*%*CI = [1.06 ~ 37.82], *p* = 0.043), FBG (odds ratio = 1.26, 95*%*CI = [1.08 ~ 1.46], *p* = 0.004), and PNI (odds ratio = 0.89, 95*%*CI = [0.81 ~ 0.99], *p* = 0.025) were the influencing factors for UTI (Supporting Information 1: Table S1), adjusted for age, inpatient days, height, urine pH, HbA1c and leucocyte‐to‐monocyte ratio. ROC curve was plotted, showing a PNI cut‐off of 42.75 (area under the ROC curve [AUC] = 0.622, *p* = 0.043) and a FBG cut‐off of 7.27 mmol/L (AUC = 0.770, *p* < 0.001), which had a good prediction effect in predicting patients who were likely to have a UTI (Figure 2).

**Table 2:** Laboratory results for patients with or without urinary tract infection.

	**Patients with UTI (** **n** = 30**)**	**Patients without UTI** ^**a**^ ** (** **n** = 106**)**	**p** ** values**
FBG (mmol/L)	12.02 ± 3.97	8.19 ± 3.57	< 0.001^†^
FBG level^b^			
< 7.27	2	54	< 0.001^†^
≥ 7.27	28	51	
HbA1c (%)	10.97 ± 2.57	9.61 ± 2.38	0.008^†^
HbA1c level			
< 7.00	2	17	0.243
≥ 7.00	28	88	
Leucocytes (10^9^/L)	6.56 ± 2.44	6.49 ± 2.03	0.842
Monocytes (10^9^/L)	0.40 ± 0.13	0.36 ± 0.14	0.081
Neutrophils (10^9^/L)	4.30 ± 2.29	4.08 ± 1.83	0.765
LMR	16.65 ± 3.94	19.09 ± 5.80	0.040^†^
CRP (mg/L)	18.91 ± 38.31	8.80 ± 27.13	0.214
PNI	47.27 ± 7.08	50.13 ± 5.35	0.018^†^
PNI level^b^			
< 42.75	9	8	0.003^†^
≥ 42.75	21	97	
Urine pH	5.57 ± 0.59	5.75 ± 0.96	0.018^†^

*Note:* Continuous variables are presented as the mean ± SD.

Abbreviations: CRP, C‐reactive protein; FBG, fasting blood glucose; HbA1c, glycosylated hemoglobin A1c; LMR, leucocyte to monocyte ratio; PNI, prognostic nutritional index; UTI, urinary tract infection.

^a^Fasting blood glucose, prognostic nutritional index, and glycosylated hemoglobin A1c level were not available for one patient without urinary tract infection.

^b^ROC curve showed a PNI cut‐off of 42.75 and a FBG cut‐off of 7.27 mmol/L to have a good predictive effect on UTI.

^†^ *p* < 0.05.

Figure 2Predictive value of prognostic nutritional index and fasting blood glucose for urinary tract infection in female patients with diabetes by ROC curve analysis. (a) Predictive value of prognostic nutritional index for urinary tract infection in female patients with diabetes by ROC curve analysis. The results indicated prognostic nutritional index as a potential predictive factor for urinary tract infection with an AUC of 0.622, 95% CI of 0.50–0.74, a cut‐off value of 42.75, a sensitivity of 30%, and a specificity of 92.4%, respectively (*p* = 0.043). (b) Predictive value of fasting blood glucose for urinary tract infection in female patients with diabetes by ROC curve analysis. The results indicated fasting blood glucose as a potential predictive factor for urinary tract infection with an AUC of 0.770, 95% CI of 0.68–0.86, a cut‐off value of 7.27 mmol/L, a sensitivity of 93.3%, and a specificity of 51.4%, respectively (*p* < 0.001).

**Figure:**
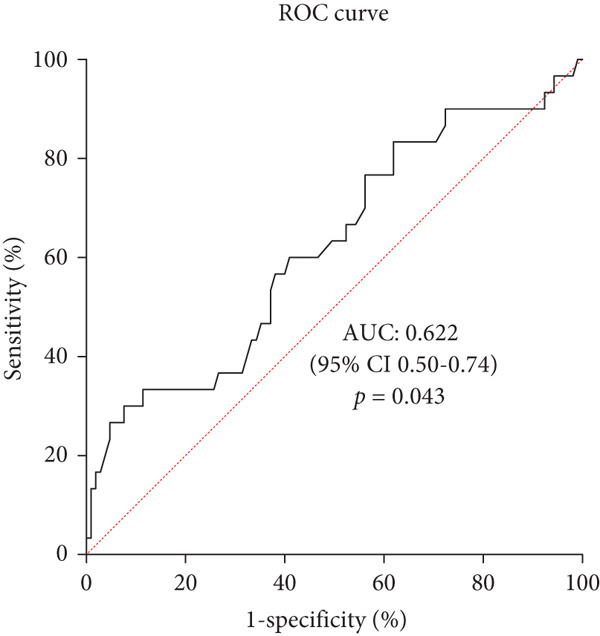
(a)

**Figure:**
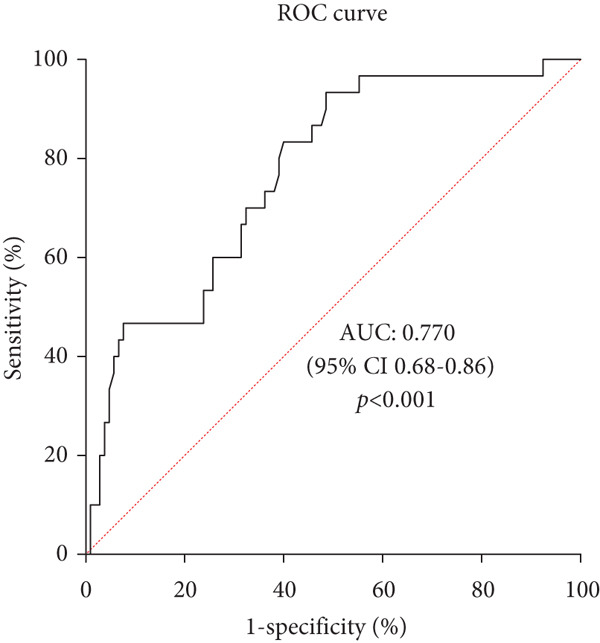
(b)

Gram‐negative bacterial isolates were detected in 21 patients with UTI, gram‐positive bacterial isolates were detected in 6 patients, and fungi were detected in 2 patients. Patients with gram‐negative bacteria had lower PNI and longer inpatient stays than those with gram‐positive bacteria (*p* = 0.045, Supporting Information 2: Table S2).

*Escherichia coli* (*E. coli*, 14/30, 46.7%) was the most predominant bacterial isolate, followed by *Klebsiella pneumoniae* (5/30, 16.7%), *Staphylococcus epidermidis* (2/30, 6.7%), and *Streptococcus agalactiae* (2/30, 6.7%, Supporting Information 4: Figure S1). Drug susceptibility testing for *Gardnerella vaginalis* was not performed due to concerns about specimen contamination. The gram‐negative bacteria were most sensitive to cefoperazone sodium/sulbactam sodium (21/21, 100.0%), imipenem (21/21, 100.0%), and ertapenem (21/21, 100.0%), with the lowest sensitivity to levofloxacin (4/21, 19.0%). The proportion of MDR in gram‐negative bacteria was 8/21 (38.1%). The gram‐positive bacteria were most sensitive to vancomycin (6/6,100.0%), linezolid (6/6, 100.0%), and tigecycline (5/5, 100.0%). The proportion of MDR in gram‐positive bacteria was 2/6 (33.3%). The susceptibility rate of levofloxacin to *E. coli* (1/14, 7.1%) was very low, as was that of *Staphylococcus epidermidis* (0/2, 0.0%) and *Streptococcus agalactiae* (0/2, 0.0%, Supporting Information 3: Table S3). Both *Candida albicans* and *Candida tropicalis* were sensitive to amphotericin B and 5‐fluorocytosine.

## 4. Discussion

In our study, the UTI prevalence in women with T2DM was 22.1%. Aamir et al. [13] found that the UTI prevalence in patients with T2DM was 8.1% (44/545), and in women, it was 13.0% (34/261). The prevalence of T2DM in women was found to be 14.2% (95% CI, 9.7%–20.2%) in a systematic review and meta‐analysis [15]. The higher UTI prevalence compared to other reports may be due to different inclusion and exclusion criteria, such as patients who had a UTI within 3 months not being excluded from this study. *E. coli* was the most common bacterial isolate, consistent with many studies, accounting for 52.3%–73.33% [13, 17–20]. Levofloxacin demonstrated high resistance to both gram‐negative and gram‐positive bacteria. Patients can obtain levofloxacin through online purchases, pharmacies, and other means, leading to the misuse and inappropriate use of levofloxacin, which is considered a reason for the high rate of levofloxacin resistance.

Married patients had a higher prevalence of UTI than nonmarried patients (including unmarried, divorced, and widowed patients). The increased sexual activity in married patients compared with nonmarried patients may explain this phenomenon. However, marital status does not reflect sexual activity and the number of sexual partners, requiring further investigation. Worku et al. [17] divided patients based on marital status: single, married, and separated/divorced. They stated that marital status was not statistically associated with bacteriuria. We redivided the patients in their study into married and nonmarried groups according to our grouping criteria, and then a *χ* ^2^ test was performed, showing that marital status was still not statistically associated with significant bacteriuria (*p* = 0.477). Based on this, we considered that the different grouping basis was not the reason for the differing results between our two studies. They included both women and men in their study, whereas our study included only women, which was considered the reason for the inconsistent results. We speculated that marital status caused a higher risk of UTI in women than in males, and the disparities in results are due to the differences in the study population.

PNI, a nutrition and inflammation‐related index, has been evaluated for its prognostic value in cancers and has confirmed its role in coronavirus disease 2019 [21–24]. To date, few studies have investigated the association between PNI and UTI. A retrospective study found that preoperative PNI level was a predictor of postoperative UTI in bladder cancer patients who underwent radical cystectomy with urinary diversion [25]. Our study found that PNI level was also a predictor of UTI in patients with T2DM, and a PNI level lower than 42.75 implied a predisposition to UTI. Interestingly, we further classified the patients with UTI according to the pathogen and found that patients with gram‐negative bacteria had a lower PNI than those with gram‐positive bacteria. The calculation of PNI involves the levels of lymphocytes and albumin. *γδ* T cell, a kind of lymphocyte, is a group of resident immune cells in the urinary tract. Mice lacking *γδ* T cell receptors are more prone to UTI than wild‐type mice [26]. Cytokines, such as IL‐1, IL‐6, IL‐8, cathelicidin, *β*‐defensin 1, and pentraxin 3, are involved in the immune defense mechanism against UTI [27]. This might explain why PNI can become a predictor of UTI in patients with Type 2 diabetes.

Some studies suggested that patients with T2DM with poor glycemic control were at an increased risk of the occurrence of UTI [18, 28, 29]. Lenherr et al. [30] enrolled 572 women with diabetes and found that a 1% increase in HbA1c was associated with a 21% increase in the probability of UTI (*p* = 0.02), after adjusting for race, hysterectomy status, urinary incontinence, sexual activity, peripheral and autonomic neuropathy, and nephropathy. Our study found that a higher level of HbA1c indicating poor glycemic control was not considered a risk factor after multivariable logistic regression analysis, consistent with previous studies [13]. However, FBG showed a predictive value for UTI in women with T2DM. We conclude that hyperglycemic states remained a risk factor. The majority of our study participants were hospitalized due to poor glycemic control, meaning that they generally had high HbA1c levels. The above phenomena may account for the nonsignificant difference in HbA1c levels between patients with and without UTI.

There were several study limitations. Our study was retrospective, and the possibility of unreliable data was unavoidable. Furthermore, confounders that were not collected such as specific drug classes (e.g., sodium–glucose cotransporter protein‐2 inhibitors), frequency of sex, and frequency of perineal scrubbing, among others, could have affected our findings. The collection of urine samples was carried out by the patients themselves at that time. Although the relevant steps had been previously preached and explained, it still could not avoid the impact of specimen contamination on the results of urine culture. The sample size needed to be further expanded, and a multicenter study should be conducted to increase the power of the results.

## 5. Conclusion

In conclusion, this study showed that PNI lower than 42.75 and FBG higher than 7.27 mmol/L may predict the occurrence of UTI in women with T2DM. Married status was also a risk factor for UTI. *E. coli* remained the predominant pathogen, and levofloxacin may no longer be appropriate for treating UTI in patients with T2DM living in this geographical area.

## Disclosure

All authors edited, reviewed, and approved the final version of the manuscript. Meng‐Die Chen is the guarantor of this work and, as such, had full access to all the data in the study and takes responsibility for the integrity of the data and the accuracy of the data analysis.

## Conflicts of Interest

The authors declare no conflicts of interest.

## Author Contributions

Chao‐Yin Lu, Qiao Liu, and Meng‐Die Chen were involved in the conception, design, conduct of the study and the analysis and interpretation of the results. Ping Feng, Han‐Ying Liu, and Yan‐Mei Shan were involved in the conduct of the study and the interpretation of the results. Chao‐Yin Lu wrote the first draft of the manuscript.

## Funding

No funding was received for this manuscript.

## Supporting Information

Additional supporting information can be found online in the Supporting Information section.

## Supporting information


**Supporting Information 1** Table S1: Association between clinical variables and urinary tract infection analyzed by multivariable logistic regression.

**Supporting Information 2** Table S2: Baseline characteristics and laboratory results for patients with gram‐negative or gram‐positive bacteria.

**Supporting Information 3** Table S3: Sensitivity analysis of pathogens to antibiotics in diabetic patients with positive urine culture.

**Supporting Information 4** Figure S1: Bacterial pathogens in diabetic patients with positive urine culture (*n* = 30).

## Data Availability

The data that support the findings of this study are available from the corresponding author upon reasonable request.
