# Supplementary material for: The Role of Prognostic Nutritional Index in UTI Susceptibility Among Female Type 2 Diabetic Patients
Source: J Diabetes Res. 2025 Dec 9;2025:6890754. doi: 10.1155/jdr/6890754 (PMC12767225; doi:10.1155/jdr/6890754)
Supplement: Supplementary file 1 — Supporting Information 1 Table S1: Association between clinical variables and urinary tract infection analyzed by multivariable logistic regression. [file JDR-2025-6890754-s003.docx]

Supplementary table 1. Association between clinical variables and urinary tract infection analyzed by multivariable logistic regression

| Clinical variables | Odds ratios | 95% CI | *P* values |
| --- | --- | --- | --- |
| Married status^*^ | 6.33 | (1.06,37.82) | 0.043^†^ |
| Age group (years old)^*^ | 0.46 | (0.15,1.45) | 0.187 |
| Inpatients stay group (days)^*^ | 1.83 | (0.48,6.90) | 0.375 |
| Height (cm) | 1.10 | (0.98, 1.22) | 0.094 |
| FBG (mmol/L) | 1.26 | (1.08, 1.46) | 0.004^†^ |
| HbA1c (%) | 1.06 | (0.83,1.36) | 0.633 |
| Peripheral blood LMR | 0.93 | (0.84, 1.03) | 0.156 |
| PNI | 0.89 | (0.81, 0.99) | 0.025^†^ |
| Urine pH | 0.64 | (0.38, 1.09) | 0.098 |

*. Married status: married patients versus unmarried patients; Age group: patients aging 55 years old or above versus patients aging below 55 years old; Inpatients stay group: patients hospitalized for 7 days or more versus those hospitalized for less than 7 days.

†. *P*<0.05

FBG: fasting blood glucose; LMR: leucocyte to monocyte ratio; HbA1c: glycosylated hemoglobin A1c; PNI: prognostic nutritional index.
