# Supplementary material for: The Role of Prognostic Nutritional Index in UTI Susceptibility Among Female Type 2 Diabetic Patients
Source: J Diabetes Res. 2025 Dec 9;2025:6890754. doi: 10.1155/jdr/6890754 (PMC12767225; doi:10.1155/jdr/6890754)
Supplement: Supplementary file 2 — Supporting Information 2 Table S2: Baseline characteristics and laboratory results for patients with gram‐negative or gram‐positive bacteria. [file JDR-2025-6890754-s001.docx]

Supplementary table 2. Baseline characteristics and laboratory results for patients with gram-negative or positive bacterial

|  | Patient with gram-negative bacterial | Patient without gram-positive bacterial | *P* values |
| --- | --- | --- | --- |
| Age (years old) | 58.23±14.16 | 54.67±7.45 | 0.175 |
| inpatients stay (days) | 9.77±3.27 | 7.00±4.15 | 0.045^*^ |
| duration of diabetes (days) | 10.79±7.00 | 15.00±4.20 | 0.112 |
| Weight (kg) | 58.50±9.70 | 59.90±11.39 | 0.723 |
| Height (cm) | 158.95±5.21 | 159.17±7.06 | 0.944 |
| BMI (kg/m2) | 23.09±3.16 | 23.46 ±2.83 | 0.800 |
| SBP (mmHg) | 135.50±18.65 | 130.33±23.52 | 0.572 |
| DBP (mmHg) | 79.68±9.16 | 76.50±16.84 | 0.538 |
| MBP (mmHg) | 98.29±10.02 | 94.44±17.99 | 0.492 |
| FBG (mmol/L) | 11.99±4.28 | 12.23±3.66 | 0.900 |
| HbA1c (%) | 10.76±2.29 | 11.15±3.75 | 0.747 |
| leucocytes (10^9^/L) | 6.56±2.72 | 5.95±1.00 | 0.849 |
| monocytes (10^9^/L) | 0.41±0.14 | 0.37±0.08 | 0.543 |
| neutrophils (10^9^/L) | 4.31±2.46 | 3.43±0.92 | 0.336 |
| LMR | 16.68±4.34 | 16.55±2.68 | 0.944 |
| CRP (mg/L) | 18.18±31.94 | 1.08±0.79 | 0.075 |
| PNI | 47.05±6.32 | 52.68±2.87 | 0.045^*^ |
| urine pH | 5.62±0.67 | 5.58±0.20 | 0.629 |

* *P*<0.05

BMI: body mass index; SBP: systolic blood pressure; DBP: diastolic blood pressure; MBP: mean blood pressure; FBG: fasting blood glucose; LMR: leucocyte to monocyte ratio; HbA1c: glycosylated hemoglobin A1c; CRP: C-reactive protein; PNI: prognostic nutritional index. Continuous variables are presented as the mean ± SD.
