# Supplementary material for: The Role of Prognostic Nutritional Index in UTI Susceptibility Among Female Type 2 Diabetic Patients
Source: J Diabetes Res. 2025 Dec 9;2025:6890754. doi: 10.1155/jdr/6890754 (PMC12767225; doi:10.1155/jdr/6890754)
Supplement: Supplementary file 3 — Supporting Information 3 Table S3: Sensitivity analysis of pathogens to antibiotics in diabetic patients with positive urine culture. [file JDR-2025-6890754-s004.docx]

Supplementary table 3. Sensitivity analysis of pathogens to antibiotics in diabetic patients with positive urine culture.

| . | *E. coli* | | | *K. pneumoniae* | | | | | *S. epidermidis* | | | *S. agalactiae* | | |
| --- | --- | --- | --- | --- | --- | --- | --- | --- | --- | --- | --- | --- | --- | --- |
|  | S^*^ | I^*^ | R^*^ | | S^*^ | I^*^ | R^*^ | S^*^ | | I^*^ | R^*^ | S^*^ | I^*^ | R^*^ |
| Cefuroxime | 5/14  (35.7%) | 0/14  (0.0%) | 9/14  (64.3%) | | 5/5  (100.0%) | 0/5  (0.0%) | 0/5  (0.0%) |  | |  |  |  |  |  |
| Cefoxitin | 10/14  (71.4%) | 0/14  (0.0%) | 4/14  (28.6%) | | 5/5  (100.0%) | 0/5  (0.0%) | 0/5  (0.0%) |  | |  |  |  |  |  |
| Cefepime | 10/14  (71.4%) | 0/14  (0.0%) | 4/14  (28.6%) | | 5/5  (100.0%) | 0/5  (0.0%) | 0/5  (0.0%) |  | |  |  |  |  |  |
| Ceftazidime | 9/14  (64.3%) | 2/14  (14.3%) | 3/14  (21.4%) | | 5/5  (100.0%) | 0/5  (0.0%) | 0/5  (0.0%) |  | |  |  |  |  |  |
| Levofloxacin | 1/14  (7.1%) | 4/14  (28.6%) | 9/14  (64.3%) | | 3/5  (60.0%) | 1/5  (20.0%) | 1/5  (20.0%) | 0/2  (0.0%) | | 0/2  (0.0%) | 2/2  (100.0%) | 0/2  (0.0%) | 0/2  (0.0%) | 2/2  (100.0%) |
| Amikacin | 14/14  (100.0%) | 0/14  (0.0%) | 0/14  (0.0%) | | 5/5  (100.0%) | 0/5  (0.0%) | 0/5  (0.0%) |  | |  |  |  |  |  |
| Cefoperazone Sodium/  Sulbactan Sodium | 14/14  (100.0%) | 0/14  (0.0%) | 0/14  (0.0%) | | 5/5  (100.0%) | 0/5  (0.0%) | 0/5  (0.0%) |  | |  |  |  |  |  |
| Piperacillin Sodium/  Tazobactam Sodium | 13/14  (92.9%) | 0/14  (0.0%) | 1/14  (7.1%) | | 5/5  (100%) | 0/5  (0.0%) | 0/5  (0.0%) |  | |  |  |  |  |  |
| Imipenem | 14/14  (100.0%) | 0/14  (0.0%) | 0/14  (0.0%) | | 5/5  (100.0%) | 0/5  (0.0%) | 0/5  (0.0%) |  | |  |  |  |  |  |
| Ertapenem | 14/14  (100.0%) | 0/14  (0.0%) | 0/14  (0.0%) | | 5/5  (100.0%) | 0/5  (0.0%) | 0/5  (0.0%) |  | |  |  |  |  |  |
| Penicillin G |  |  |  | |  |  |  | 1/2  (50.0%) | | 0/2  (0.0%) | 1/2  (50.0%) | 2/2  (100.0%) | 0/2  (0.0%) | 0/2  (0.0%) |
| Tigecycline |  |  |  | |  |  |  | 2/2  (100.0%) | | 0/2  (0.0%) | 0/2  (0.0%) | 2/2  (100.0%) | 0/2  (0.0%) | 0/2  (0.0%) |
| Moxifloxacin |  |  |  | |  |  |  | 0/2  (0.0%) | | 1/2  (50.0%) | 1/2  (50.0%) | 0/2  (0.0%) | 0/2  (0.0%) | 2/2  (100.0%) |
| Erythromycin |  |  |  | |  |  |  | 0/2  (0.0%) | | 0/2  (0.0%) | 2/2  (100.0%) | 0/2  (0.0%) | 0/2  (0.0%) | 2/2  (100.0%) |
| Vancomycin |  |  |  | |  |  |  | 2/2  (100.0%) | | 0/2  (0.0%) | 0/2  (0.0%) | 2/2  (100.0%) | 0/2  (0.0%) | 0/2  (0.0%) |
| Linezolid |  |  |  | |  |  |  | 2/2  (100.0%) | | 0/2  (0.0%) | 0/2  (0.0%) | 2/2  (100.0%) | 0/2  (0.0%) | 0/2  (0.0%) |

*. S: susceptible; I: intermediate; R: resistant.

*Citrobacter freundii, Proteus mirabilis, Staphylococcus aureus*, *Enterococcus faecalis,* *Candida albicans* and *Candida tropicalis* were only detected in one patient and sensitivity analysis of theses pathogens were not displayed in the table.

*Escherichia coli: E. coli; Klebsiella* *pneumoniae: K. pneumoniae; Staphylococcus epidermidis: S. epidermidis; Streptococcus agalactiae: S. agalactiae.*
