# Supplementary material for: The Role of Prognostic Nutritional Index in UTI Susceptibility Among Female Type 2 Diabetic Patients
Source: J Diabetes Res. 2025 Dec 9;2025:6890754. doi: 10.1155/jdr/6890754 (PMC12767225; doi:10.1155/jdr/6890754)
Supplement: Supplementary file 4 — Supporting Information 4 Figure S1: Bacterial pathogens in diabetic patients with positive urine culture (n = 30). [file JDR-2025-6890754-s002.pdf]

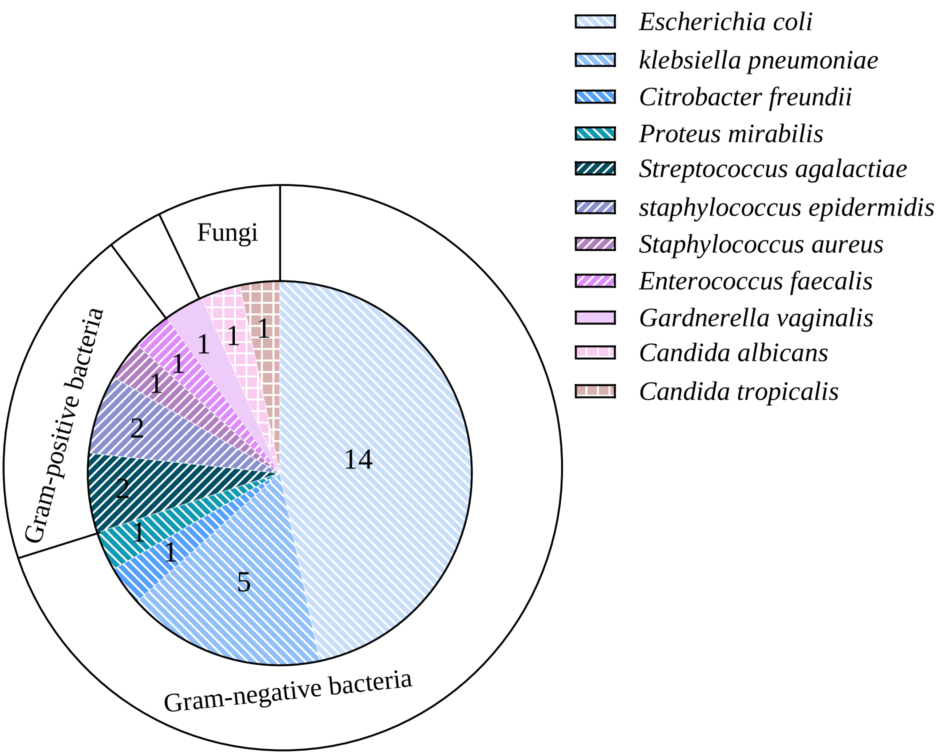

Supplementary figure 1. Bacterial pathogens in diabetic patients with positive urine culture (n = 30)
